# Time-resolved cryogenic electron tomography for the study of transient cellular processes

**DOI:** 10.1091/mbc.E24-01-0042

**Published:** 2024-07-01

**Authors:** Joseph Yoniles, Jacob A. Summers, Kara A. Zielinski, Cali Antolini, Mayura Panjalingam, Stella Lisova, Frank R. Moss, Maximus Aldo Di Perna, Christopher Kupitz, Mark S. Hunter, Lois Pollack, Soichi Wakatsuki, Peter D. Dahlberg

**Affiliations:** a Biophysics Program, Stanford University School of Medicine, Stanford, CA 94305; b Department of Structural Biology, Stanford University School of Medicine, Stanford, CA 94305; c School of Applied and Engineering Physics, Cornell University, Ithaca, NY 14853; d Stanford Synchrotron Radiation Lightsource, SLAC National Accelerator Laboratory, Menlo Park, CA 94025; e Linac Coherent Light Source, SLAC National Accelerator Laboratory, Menlo Park, CA 94025; f Department of Chemistry, New York University, New York, NY 10003; g Department of Electrical Engineering, Stanford University, Stanford, CA 94305; Harvard University

## Abstract

Cryogenic electron tomography (cryo-ET) is the highest resolution imaging technique applicable to the life sciences, enabling subnanometer visualization of specimens preserved in their near native states. The rapid plunge freezing process used to prepare samples lends itself to time-resolved studies, which researchers have pursued for in vitro samples for decades. Here, we focus on developing a freezing apparatus for time-resolved studies in situ. The device mixes cellular samples with solution-phase stimulants before spraying them directly onto an electron microscopy grid that is transiting into cryogenic liquid ethane. By varying the flow rates of cell and stimulant solutions within the device, we can control the reaction time from tens of milliseconds to over a second before freezing. In a proof-of-principle demonstration, the freezing method is applied to a model bacterium, *Caulobacter crescentus,* mixed with an acidic buffer. Through cryo-ET we resolved structural changes throughout the cell, including surface-layer protein dissolution, outer membrane deformation, and cytosolic rearrangement, all within 1.5 s of reaction time. This new approach, Time-Resolved cryo-ET (TR-cryo-ET), enhances the capabilities of cryo-ET by incorporating a subsecond temporal axis and enables the visualization of induced structural changes at the molecular, organelle, or cellular level.

## Abstract (executive summary)

Capturing cellular dynamics with millisecond precision and nanometer-scale spatial resolution is an open challenge and of critical importance due to the many cellular processes that occur on these time and length scales.

The sample preparation method presented here enables the rapid mixing of cellular samples with solution-phase stimulants followed by rapid freezing at well-controlled time points.

Numerous methods of interrogation can be performed following freezing, including cryogenic electron tomography, which enables the visualization of dynamic cellular processes with unprecedented simultaneous spatial and temporal resolution.

## INTRODUCTION

There are three main varieties of cryogenic electron microscopy (cryo-EM) for biological samples: single-particle cryo-EM (Elmlund and Elmlund, 2015; Cheng, 2018; Lyumkis, 2019), microcrystal electron diffraction (Nannenga and Gonen, 2021; Saha *et al.*, 2022), and cryogenic electron tomography (cryo-ET) (Asano *et al.*, 2016; Plitzko and Baumeister, 2019; Turk and Baumeister, 2020). While the samples for these three approaches often differ in composition–whether they involve purified biomolecules in solution, crystallized biomolecules, or intact cells/cell fragments, respectively–they all undergo a common step in sample preparation: rapid freezing. This rapid freezing is most commonly achieved by “plunge freezing,” where the aqueous sample is deposited on an electron microscopy grid before being plunged into a cryogenic liquid, typically liquid ethane or an ethane-propane mixture. Plunge freezing cools samples that are less than ∼10 µm in thickness so rapidly that amorphous rather than crystalline ice is formed (Dobro *et al.*, 2010). The requisite cooling rates for this process are on the order of 10^4^–10^5^ K/s, resulting in a transition from room temperature to cryogenic temperatures within roughly one millisecond (Bikker, 2019; Voss *et al.*, 2021). It was recognized early on during the development of cryo-EM that the obligatory rapid freezing lends itself well to time-resolved studies (Berriman and Unwin, 1994; Unwin, 1995), leading to an approach known as time-resolved cryo-EM (TR-cryo-EM). TR-cryo-EM is an active and exciting area of research where structural dynamics are initiated either through rapid mixing (Feng *et al.*, 2017; Kaledhonkar *et al.*, 2019; Dandey *et al.*, 2020; Bhattacharjee *et al.*, 2024) or light activation (Yoder *et al.*, 2020) before freezing. However, to date these studies have been restricted to single-particle cryo-EM experiments involving purified biomolecules. Here, we extend the approach of TR-cryo-EM to electron tomography to study the transient processes of cellular systems in situ*.*

## RESULTS AND DISCUSSION

**FIGURE 1:**
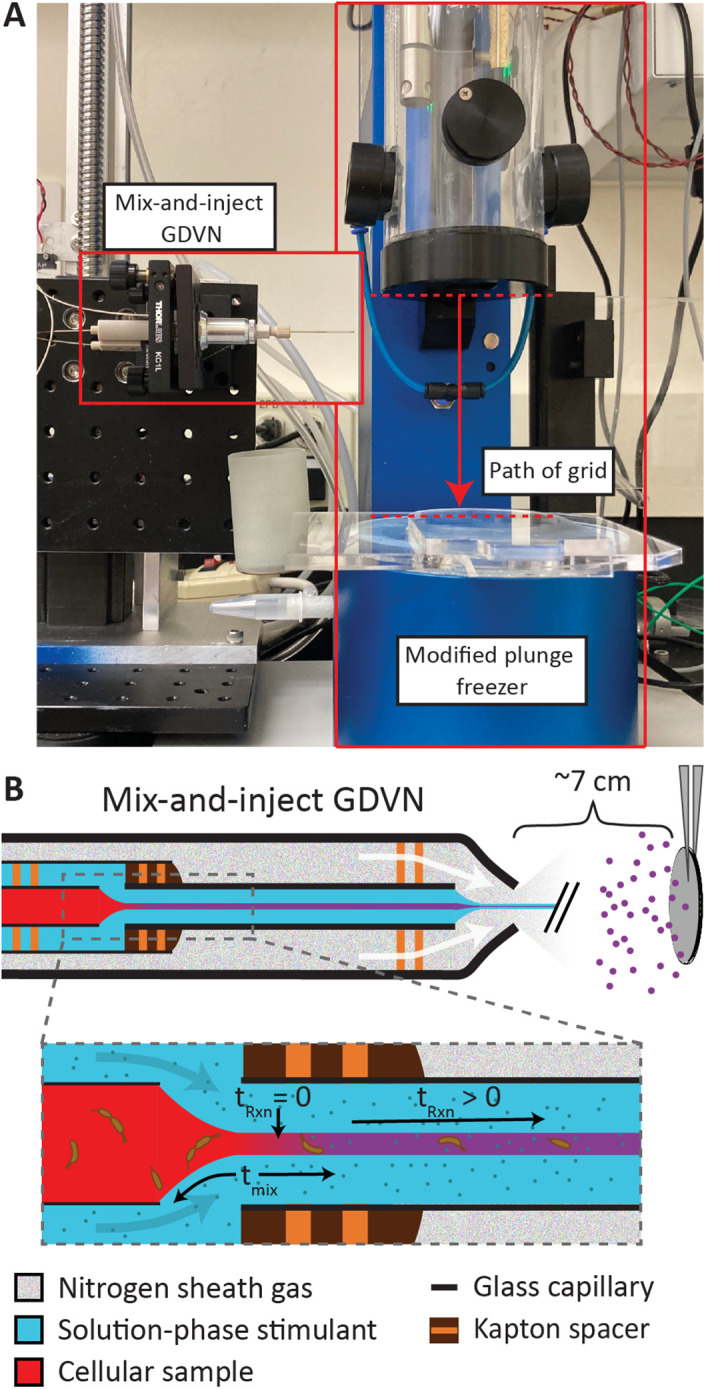
Overview of apparatus for time-resolved freezing. (A) Picture of the setup with the microfluidic mix-and-inject GDVN (mixer) on the left and plunge freezer on the right. The mixer generates an aerosol that the electron microscopy grid transits through on its path to cryogenic liquid ethane. The region where the grid can interact with the aerosol is marked by dashed red lines. (B) Cartoon view of the inner workings of the mixer. Zoomed region highlights the hydrodynamic focusing of the cellular sample which results in a rapid diffusion-based mixing over a timescale given by t_mix_.

The apparatus, shown in Figure 1 and Supplemental Figure S1, consists of a microfluidic device that generates an aerosol of stimulated cells that are incident on a grid transiting into liquid ethane. The fluidic device is known as a mix-and-inject Gas Dynamic Virtual Nozzle (GDVN) and is routinely used for time-resolved crystallography, where the reaction of microcrystal samples with solution-phase stimulants occurs before the injection into the path of an x-ray free electron laser (Olmos *et al.*, 2018; Calvey *et al.*, 2019, 2020; Pandey *et al.*, 2021). This microfluidic device, hereafter referred to as a “mixer,” consists internally of a series of concentric capillaries. The innermost capillary contains the cellular sample of interest, which is surrounded by another capillary that carries the solution-phase stimulant. The cells and stimulant first interact in a focusing region formed by a small gap between two capillaries where the outer sheath flow thins the central cellular sample stream in a process known as hydrodynamic focusing, see Figure 1B. The thin width of the sample stream enables rapid diffusion of the stimulant into the stream. The diffusive mixing time, t_mix_, depends on the geometry of the mixer, the flow rates chosen (which sets the central sample stream width), the ratio between these flow rates, and most critically, the size of the stimulant. Mixing times on the single millisecond scale are achievable, but uncertainty from the subsequent delay after mixing can vary on the order of sub millisecond to tens of milliseconds. For timepoints on the hundreds of millisecond scale, like in this study, the total uncertainty from the mixer, including both mixing and delay, is on the order of tens of milliseconds and while brief, dominates the uncertainty of the overall reaction time (t_Rxn_), see *Materials and Methods* and Supplemental Figure SI for more details. In general, mixers must be customized for each system to match the time range of experimental interest while considering other practicalities, such as sample consumption and device performance.

The dynamics of interest proceed as the mixed solution flows down the length of the capillary before expulsion from the device using an inert gas such as helium or nitrogen. In typical applications of the mixer, the goal is to create a free-standing liquid column (jet) just outside the tip of the device, however, for our application it was desirable to create an aerosol, as the direct jet hitting the grid would have damaged the fragile support film. Droplets from this aerosol are heterogenous in size (Supplemental Figures S2 and S3) but are on the order of micrometers in diameter. Using a commercial plunge freezer (Gatan CP3) with minimal modification, these droplets hit and adhere to a hydrophilic electron microscopy grid on transit into liquid ethane. When the electron microscopy grid enters the ethane, the sample is rapidly frozen and the dynamics are arrested. At a later time, convenient to the researcher, the sample can be loaded into an electron microscope for cryo-ET data collection.

The reaction time is defined as the duration from the moment the sample starts mixing (refer to *Materials and Methods*) to the moment at which the sample freezes and the dynamics come to a halt. This process involves five distinct steps: the initial mixing process, the time for the sample to flow through the mixer device postmixing, the time-of-flight of the aerosol, the transit time of the grid to the ethane after the aerosol deposition, and finally, the freezing time required to bring the sample to cryogenic temperatures upon submersion in liquid ethane.

**FIGURE 2:**
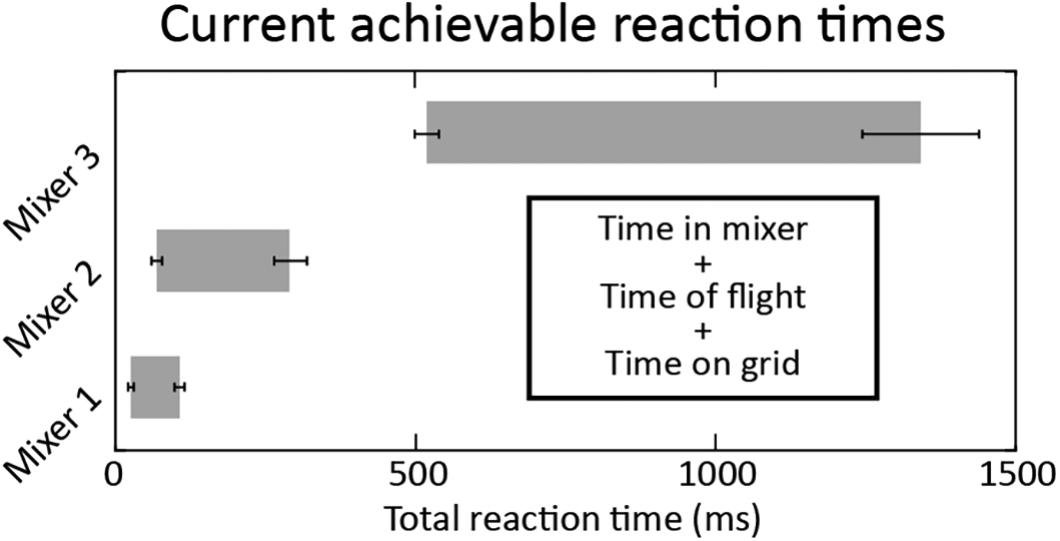
Range of accessible reaction times across different mixer geometries. These ranges reflect the total reaction time, including the variable time on grid before freezing. The study detailed below used only mixers 2 and 3 because the dynamics were covered well by those mixers. The lack of overlap between the achievable reaction times of mixers 2 and 3 is a consequence of mixer geometry design, and not a physical or technical limitation of the method. Additional mixers could fill this gap.

The first two steps are coupled and are controlled by the geometry of the mixer and the flow rates of sample and stimulant. During an experiment, the sample and stimulant are loaded into separate fluidic reservoirs. HPLC pumps are then used to push the fluid out of the reservoirs at controlled flow rates (see Supplemental Figure S1). For a single mixer geometry, different reaction times can be achieved by changing the sample and stimulant flow rates, but flow rates of both the sample and stimulant have a maxima and minima to ensure good device performance and a desirable density of droplet deposition. If the flow rates are too low, there is too little material deposited on the electron microscopy grid as it transits through the aerosol. If the flow rates are too high, the droplet size is too large to provide suitably thin ice for subsequent electron tomography. Furthermore, pressures required to generate such high flow rates may lead to mechanical failure of the fittings or HPLC pumps. Critically, the ratio of the sample flow rate to the stimulant flow rate sets the inner stream width and thus, mixing time and associated uncertainty (see Supplemental Figure SI for further details). Lower sample flow rates produce thinner streams and lead to less uncertainty, however, the sample flow rate also influences the final density of cells on the EM grid, with higher relative sample flow rates resulting in better deposition, so the flow rates are carefully selected to balance the uncertainty and the final cell concentration. In practice, total flow rates were varied from 35 – 130 µl/min and the ratio of stimulant to sample was kept between 1.5 and 5.8. To achieve a broad range of reaction times, multiple mixers with different geometries must be employed (see Figure 2 and Supplemental Table S1).

The third step, the time-of-flight of the aerosol droplets, is a function of the sheath gas pressure, total liquid flow rate, mixer nozzle diameter, and distance from the nozzle tip to the grid. The distance to the grid and the sheath gas pressure was held constant across mixers so the velocity of the aerosol could be characterized for each mixer (see Supplemental Figure S3 and Supplemental Table S1). An upper limit to the time-of-flight was found to be 1–4 ms based on previous jet velocity measurements. This travel time is small, but not negligible, and is consistent with velocity measurements from similar devices (DePonte *et al.*, 2008). The fourth step, the time on grid postdeposition, is determined by the mechanics of the plunge freezer, with the longest time delay limited by the line of sight between the mixer and the grid at the top of the plunging stroke, and the shortest time delay limited by the line of sight to the grid at the bottom of the plunging stroke. In practice, this time range is 16–89 ms. The current device is gravity-driven; future modifications could alter the geometry or control the driving force for the plunging mechanism to modulate this time window. Lastly, the sample freezing time is determined by sample thickness. Experimentally determining the time needed to arrest dynamics is difficult, but with micron-diameter droplets, all indications are that freezing occurs on the order of a single millisecond and is negligible compared with the other contributions to the reaction time. See Supplemental Table S1 and Supplemental text for a detailed discussion of the uncertainties associated with each of these steps.

**FIGURE 3:**
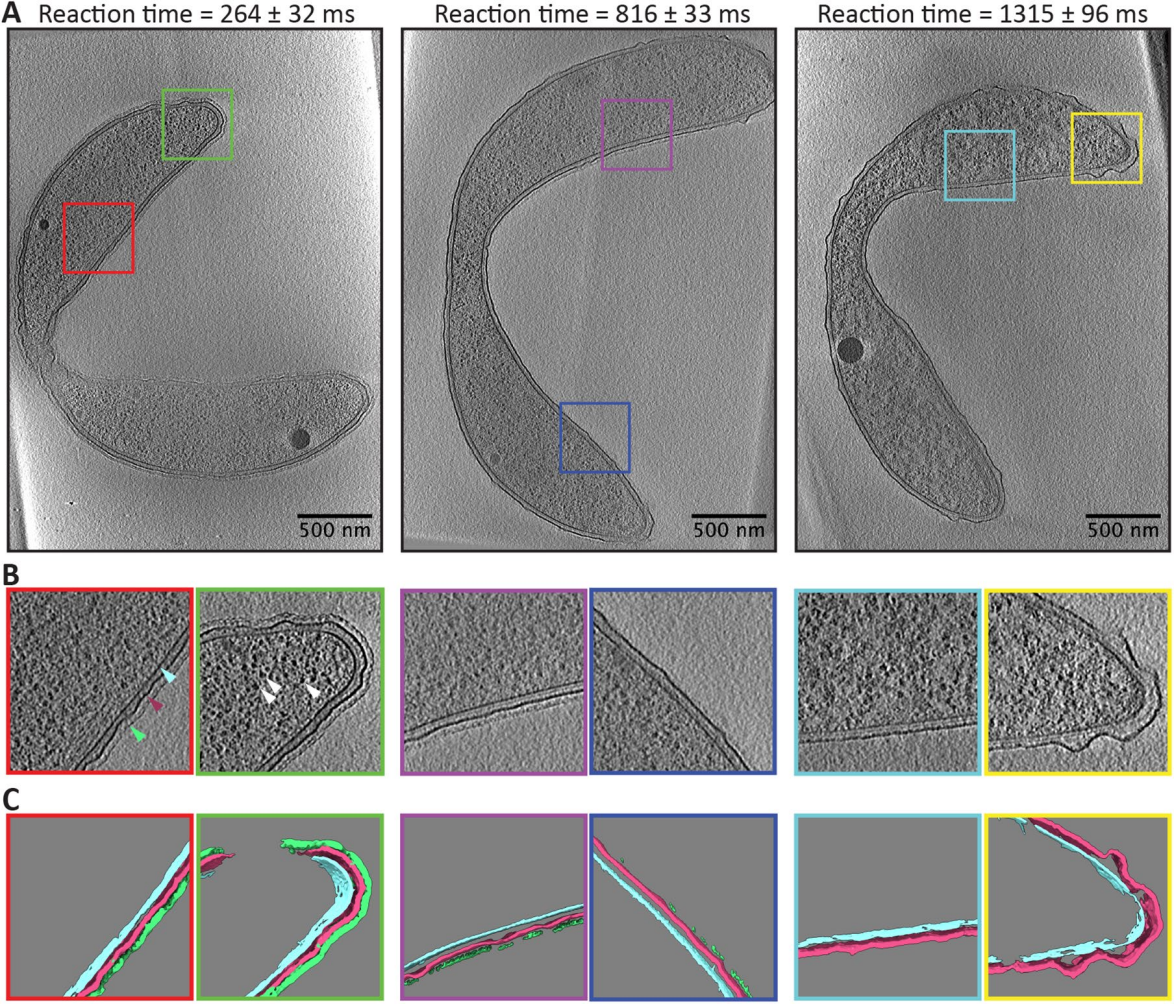
Representative tomography data from time-resolved pH jump. (A) Single slices from the tomographic reconstruction of a representative cell frozen at the listed reaction times. Colored squares highlight zoomed in regions shown in (B) where the S-layer (green pointer), outer membrane (maroon pointer), inner membrane (cyan pointer), and ribosomes (white pointers) are clearly visible. (C) Segmentation of the central 50 slices of the tomographic reconstructions shown in B. The segmentation highlights the S-layer (green), the outer membrane (maroon), and inner membrane (cyan). These segmentations from the central slices were used for subsequent data analysis.

As a proof-of-principle demonstration, we performed a pH jump experiment on the Gram-negative freshwater bacterium *Caulobacter crescentus.* Like many bacteria and archaea, *C. crescentus* possesses a pseudocrystalline surface layer of proteins known as the S-layer. In *C. crescentus*, the S-layer is made entirely of the protein RsaA, which forms a hexameric lattice over the surface of the bacterium (Comerci *et al.*, 2019; Herrmann *et al.*, 2020). RsaA assumes a folded form near neutral pH and in the presence of calcium, but denatures at acidic pH (Walker *et al.*, 1992). The S-layer is also readily visible when imaged by cryo-ET (Bharat *et al.*, 2017). Using the time-resolved freezing apparatus described above, we mixed *C. crescentus* cells (grown in standard media) with HEPES buffer at pH 2.0. In total, grids were frozen with the following reaction times: t_Rxn_ = 264, 567, 816, 1066, and 1315 ms. Cells were also frozen after mixing with their own media rather than the HEPES buffer as a control. Following freezing, standard cryo-ET data acquisition and tomographic reconstruction were performed, see *Materials and Methods*. These control cells exhibited comparable structural features to previously published cryo-ET reconstructions of *C. crescentus* (Briegel *et al*., 2006, 2008; Bharat *et al*., 2017; Dahlberg *et al*., 2020), suggesting that spray freezing maintains the structural integrity of the cell and subcellular features to a degree that is not significantly different from traditional plunge freezing (see Supplemental Figure S4). Figure 3 shows representative tomography data collected on cells from various reaction times that were in a predivisional state of the cell cycle, hence the narrowing of the cell body (see Supplemental Video). The grayscale tomography data was subsequently segmented to highlight known structural features, in this case the S-layer, outer membrane, and inner membrane.

**Figure:** Movie S1

The outermost layer of the cell, the S-layer, is seen as a pseudocrystalline lattice with lower contrast than the outer membrane. The expected removal of the S-layer with reaction time was observed, with almost all S-layer being gone after 1 s. We also consistently observed two other phenomena: the ruffling of the outer membrane and a change in the appearance of the cytoplasm (primarily noticeable as a change in the contrast between ribosomes and the rest of the cytosol). The loss of the S-layer and ruffling of the outer membrane are straightforward to quantify and are done so here, but quantification of the change in the cytoplasm is beyond the scope of this work.

**FIGURE 4:**
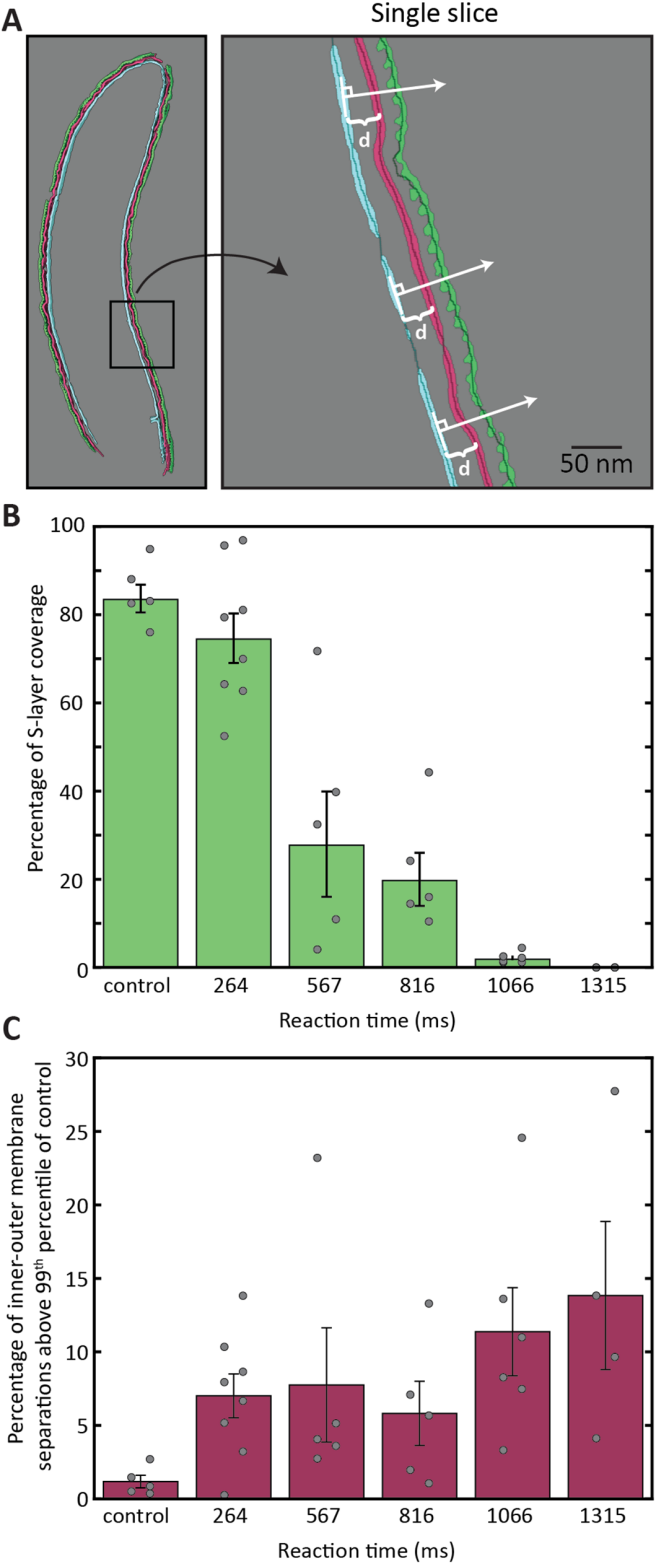
Quantification of S-layer removal and outer-membrane ruffling. (A) Cartoon demonstration of the method for calculating s-layer removal and membrane ruffling that uses normal vectors from the inner membrane to calculate membrane separation and the presence or absence of S-layer. See text for more details. (B) Percentage of cell surface covered by S-layer for each time point (colored bars). Circles show single-cell measurements and error bars represent the standard error of the mean of single-cell measurements. (C) Same as B, except quantification of membrane ruffling. See text for further details.

The inner membrane remained consistently smooth across cells in all reaction times, so it served as a basis for quantifying S-layer presence and outer membrane ruffling. For this analysis, the inner membrane, outer membrane, and S-layer segmentations were all thinned to single-pixel curves, see *Materials and Methods* for more details. A normal vector pointing to the extracellular space was calculated at each pixel in the inner membrane curve, see Figure 4A. The S-layer coverage was determined by whether the S-layer was intersected by the normal vector. Similarly, the distance between inner and outer membrane was calculated by the point of intersection with the normal vector.

Interestingly, one can see that the S-layer removal (quantified in Figure 4B) does not occur at a constant rate. The control cells – those that were mixed with their own media before freezing – show a high percentage of S-layer coverage at 83%. The majority of the deviation from 100% coverage is likely due to errors in the segmentation process, as the segmentation of the dense, uniformly present outer membrane yielded 87% coverage across all reaction times. The 264-ms time point shows a marginal decrease in S-layer coverage, with an increased spread in single-cell measurements. The 567-ms timepoint shows a large drop in the average S-layer coverage, but also a significant spread in coverage from cell to cell, ranging from 4–72%. As the reaction time progresses, the spread in cell measurements decreases, as does the average coverage of S-layer. Our interpretation of this result is that the S-layer fails catastrophically, falling off in large sheets. This conclusion is consistent with our tomography data of partially covered cells where S-layer coverage exhibited both large sections of intact S-layer and large sections of bare outer membrane, see Figure 3 reaction time 816 ms.

Turning now to the quantification of the outer membrane ruffling. This ruffling is best represented by the proportion of intermembrane distance separations that could be categorized as extreme, see Supplemental Figure S5. The control cells were used to determine the threshold of separations that would be considered extreme, choosing the 99 th percentile as this threshold, *d* = 20.4 nm. Surprisingly, there is a jump in the intermembrane separation present at the shortest timepoint of 264 ms. This jump largely precedes the S-layer removal, suggesting that the S-layer loss may be related to loss of outer membrane integrity in addition to the pH change.

This work demonstrates the ability of a new sample preparation method for time-resolved cryogenic electron tomography (TR-cryo-ET) to capture ultrastructural changes in situ with nanometer scale spatial resolution and better than 25 millisecond temporal resolution in bacterial systems. The approach provides much needed temporal context to the snapshots provided normally by cryo-ET, expanding the types of questions that can be addressed by cryo-ET and opening new avenues of microbiology research. Future work will explore the range of samples that can be frozen in this manner and will extend the stimulation to include optical stimulation. Importantly, we highlighted the use of cryo-ET with this sample preparation device, but any number of methods could be employed with this instrument, including soft x-ray tomography (Kounatidis *et al.*, 2020) and cryogenic fluorescence microscopy. Future work will incorporate more advanced workflows such as focused ion beam milling (Rigort *et al.*, 2012) and advanced methods of cryogenic correlative light and electron microscopy such as super-resolution and fluorescent biosensors (Dahlberg and Moerner, 2021; Perez *et al.*, 2022).

## MATERIALS AND METHODS

### Cell preparation

*C. crescentus* cells of the strain NA1000 were cultured following established protocols in PYE media to the late log phase (Poindexter, 1964). Before freezing, cells were concentrated by centrifugation by ∼10x to increase the number of cells deposited on the electron microscopy grid.

### Grid pretreatment

Continuous carbon finder grids (EMS Catalog # cf-200-cu-50) were pretreated with 15-nm gold beads (EMS Catalog # 25489) by drop casting. By pretreating the grids with fiducials, there was no need to aerosolize a solution of nanoparticles. Before plunge freezing, the grids were plasma etched for 15 s at 15 mA.

### Mixer fabrication and operation

Mixers were fabricated as previously described (Calvey *et al.*, 2016, 2019). Briefly, a supply line (Polymicro Technologies, Phoenix, AZ; 50–100 μm inner diameter, 200-μm outer diameter) is polished and beveled. Centering spacers, laser cut out of a Kapton sheet, are placed on the tip. A second piece of capillary, known as the delay line, is similarly polished and beveled, and then glued inside a larger piece of glass. The supply line is inserted into the same piece of glass directly upstream of the delay line. Standard fittings (IDEX Health and Science, West Henrietta, NY) are used to secure the supply line ∼75 μm away from (upstream of) the start of the delay line, which creates the focusing region. Lastly, a glass nozzle is flame polished to create an opening ∼80–110 μm in diameter. The mixer is inserted into this larger nozzle, which is then positioned to optimize jetting.

For grid making, HPLC pumps (LC-20AD from Shimadzu Scientific Instruments) are used for liquid delivery and nitrogen gas is used to accelerate the liquid out of the nozzle to create a spray. To produce a spray, instead of a jet that has been used for previous applications of these mixing injectors, the flow rate of the nitrogen gas is increased from the typical range of 30–40 mg/min used for jetting to at least 70 mg/min. Water is first flowed through all lines to establish a stable spray. The nozzle is mounted on a 3-axis stage and its position is adjusted to ensure alignment with the grid. Then, a shutter is placed in front of the nozzle to avoid prewetting the grid as it is mounted. Next, the liquid stimulant (low pH buffer) and the sample (cells) are loaded into separate reservoirs (Neptune Fluid Flow Systems, LLC., Knoxville, TN). The appropriate flow rates are set for the timepoint of interest and the lines are flushed for ∼3 min. Then, the tweezers, with the grid, are mounted onto the plunge freezer arm. The shutter is removed, and the grid is quickly plunged through the stream to accumulate the freshly mixed sample. The shutter is put back in place and the grid is transferred to a storage box. Different timepoints can be rapidly acquired by simply changing the flow rates, or by changing nozzles optimized for a different range of timepoints.

### Cryo-ET data collection and segmentation

Cryo-ET data were collected using a 300 keV electron microscope (Titan Krios ThermoFisher) with a direct detection camera (K3 Gatan) and an energy filter (BioquantumGatan). The tilt series was acquired with 2° steps in a bidirectional (Hagen *et al.*, 2017) manner beginning with the sample at –30° and extending to ±60°. The total dose for each tilt series was 120 e-/Å^2^. Tilt series were reconstructed using the Etomo package in IMOD (Kremer *et al.*, 1996). Tilts were acquired with an effective pixel size of 6.45 Å and 10 μm of defocus. These imaging parameters were chosen so that entire *C. crescentus* cells could be acquired in a single tomogram. The resulting tomograms were binned by two and features were then segmented with the assistance of a neural network implemented in EMAN2 (Chen *et al.*, 2017). For visualization in the manuscript figures, the tomographic reconstructions were low-pass filtered in three dimensions with a Gaussian kernel with a sigma of three binned pixels. Statistics on the total number of tilt series collected and the number of reconstructions used for analysis are given in Supplemental Table S2.

### Cryo-ET quantification

S-layer presence and outer membrane ruffling were quantified with identical methods. The inner membrane, outer membrane, and S-layer were segmented with EMAN2 and processed in a custom MATLAB script. To minimize cellular curvature artifacts in the intermembrane distance measurement, the 50 middle slices of the cell were extracted for analysis. The inside and outside of cells were manually cleaned, and intensity thresholds were selected such that the segmentation was representative of the cellular feature within each tomogram. To facilitate the generation of normal vectors across the inner membrane, the membrane and S-layer segmentations were thinned to single-pixel thick curves in each two-dimensional slice using the built-in “bwmorph” function. Extracellular-facing normal vectors for each inner membrane pixel were calculated by considering neighboring pixels of up to 10 pixels away. These normal vectors were drawn on each inner membrane pixel and intersections with S-layer and outer membrane pixels were recorded.

Careful consideration was required when quantifying outer membrane ruffling. Single-pixel thick normal vectors were initially generated to intersect with the outer membrane, but in many cases, this vector would visually intersect with the outer membrane without pixels explicitly overlapping. To address these situations, three-pixel thick normal vectors were generated, and the average distance to multiple outer membrane pixels was recorded. Measurements including more than four outer membrane pixels were deemed noisy and discarded. We anticipated that outer membrane ruffling would be quantified by a change in the SD of the distance distributions in Supplemental Figure S5, meaning that the intermembrane distance should have shrunk and grown as a function of reaction time. However, the ruffling phenomenon appears to only increase the intermembrane separation. Therefore, outer membrane ruffling is best represented by the relative increase in extreme intermembrane distances, shown in Figure 4C. For this calculation, we first determined the intermembrane distance of the control sample (top left of Supplemental Figure S5) which corresponds to 99 th percentile of the distance distribution, 20.4 nm. We then integrated the histograms of different reaction times above this intermembrane distance threshold.

## Supplementary Material



## Abbreviations used:

cryo-EM: cryogenic electron microscopy
cryo-ET: cryogenic electron tomography
GDVN: gas dynamic virtual nozzle
TR-cryo-EM: time-resolved cryogenic electron microscopy
TR-cryo-ET: time-resolved cryogenic electron tomography.
