# Supplementary material for: Time-resolved cryogenic electron tomography for the study of transient cellular processes
Source: Mol Biol Cell. 2024 Jul 1;35(7):mr4. doi: 10.1091/mbc.E24-01-0042 (PMC11244162; doi:10.1091/mbc.E24-01-0042)
Supplement: Supplementary file 2 [file mbc-35-mr4-s001.pdf]

Supplemental Materials

Molecular Biology of the Cell

Yoniles *et al*.

Supplementary Material: Time-resolved cryogenic electron tomography for the study of transient cellular processes

Joseph Yoniles[1]*, Jacob A. Summers[2]*, Kara A. Zielinski[3], Cali Antolini[4,5], Mayura Panjalingam[6], Stella Lisova[5], Frank R. Moss III[5], Maximus Aldo Di Perna[7], Christopher Kupitz[5], Mark S Hunter[5], Lois Pollack[3], Soichi Wakatsuki[2,4], Peter D. Dahlberg[4]

[1]Biophysics Program, Stanford University School of Medicine, Stanford, CA, USA

[2]Department of Structural Biology, Stanford University School of Medicine, Stanford, CA, USA

[3]School of Applied and Engineering Physics, Cornell University, Ithaca, NY, USA

[4]Stanford Synchrotron Radiation Lightsource, SLAC National Accelerator Laboratory, Menlo Park, CA, USA

[5]Linac Coherent Light Source, SLAC National Accelerator Laboratory, Menlo Park, CA, USA

[6]Department of Chemistry, New York University, New York, New York, USA

[7]Department of Electrical Engineering, Stanford University, Stanford, CA, USA

Supporting Figures

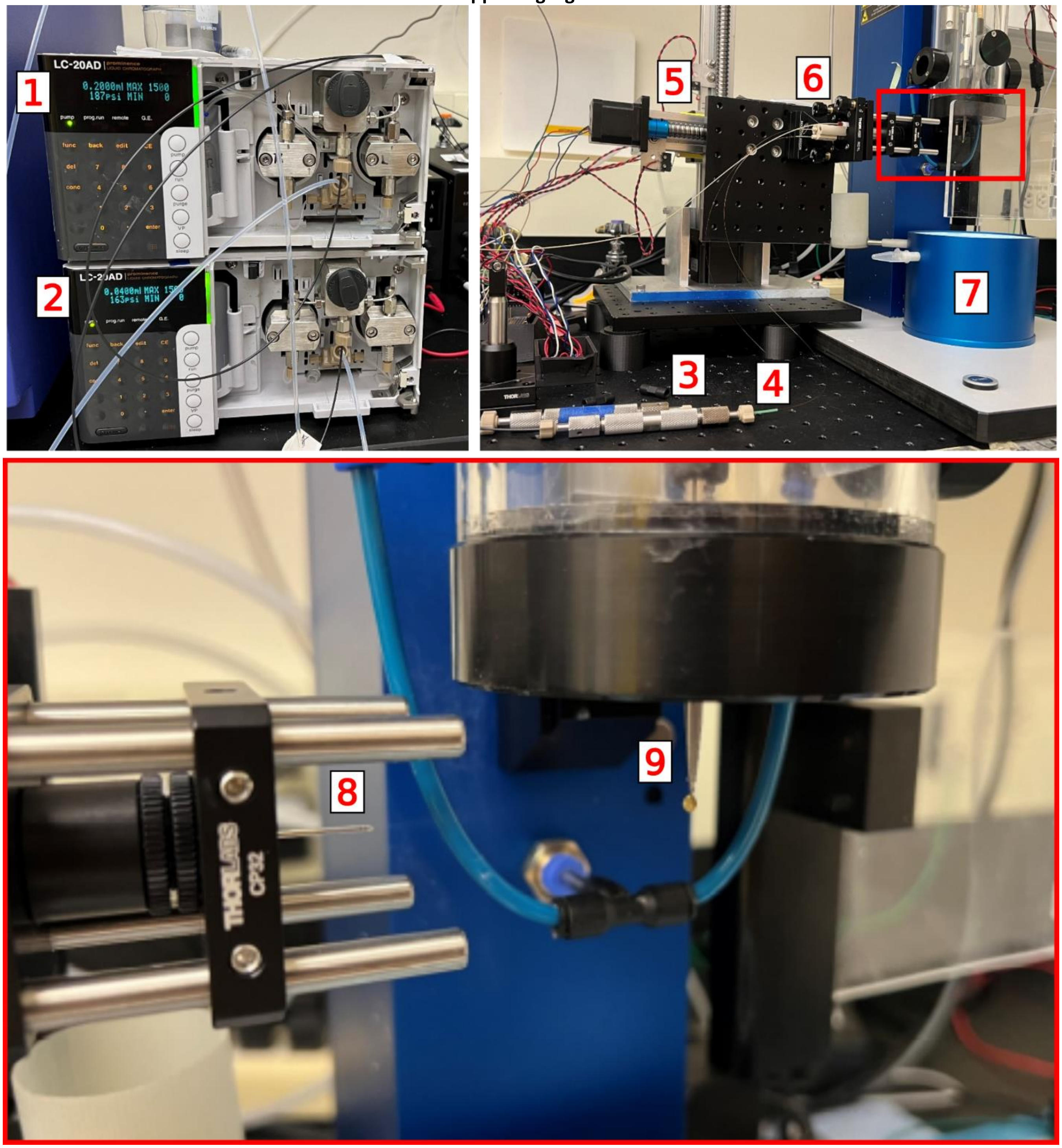

Figure S1: Detailed schematic of the freezing apparatus. The stimulant and sample HPLC pumps (1 & 2) generate fluid flow from the stimulant and sample reservoirs (3 & 4). A mechanical stage (5) supports the GDVN (6), where the stimulant and sample are delivered with precise flow rates and mixed for well-defined reaction times. Interfaced with a Gatan CP3 plunge freezer (7), the GDVN nozzle (red inset, 8) aerosolizes the mixed sample onto a continuous carbon electron microscopy grid (9) transiting through the aerosol and falling into liquid ethane.

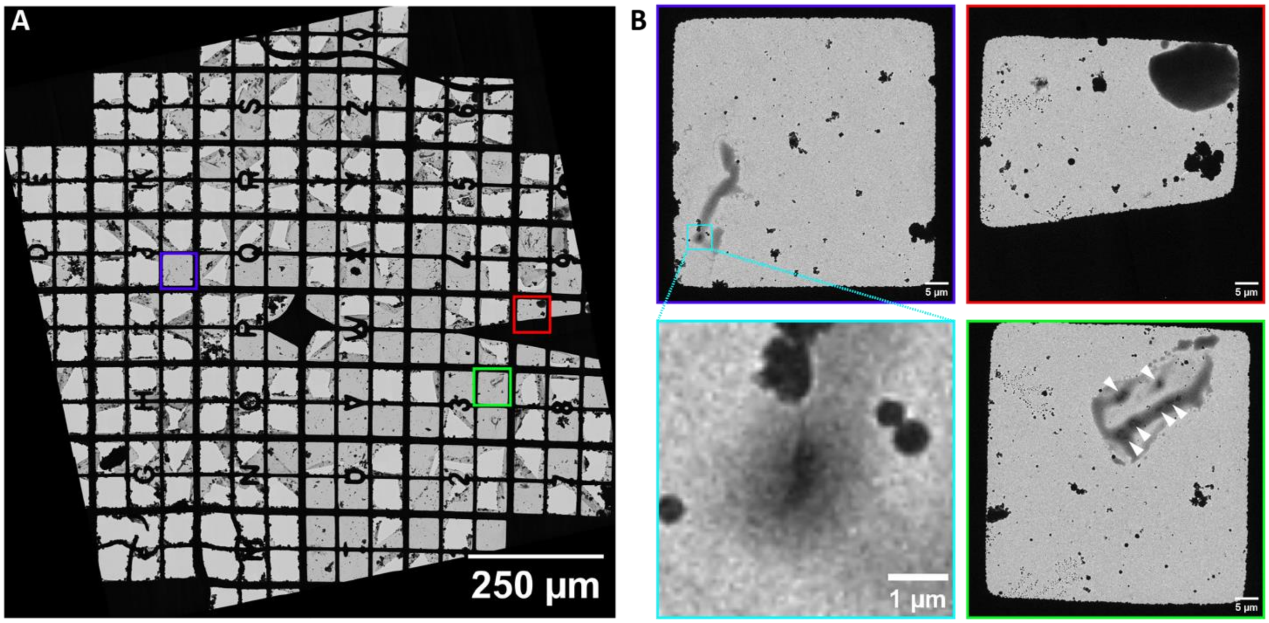

Figure S2: Heterogeneous droplets obtained by spray-freezing bacterial samples of *C. crescentus*. (A) Low magnification montage of the electron microscopy grid. The continuous carbon support film is quite fragile leading to numerous broken squares. Colored boxes highlight zoomed regions in B. (B) Various droplets shown in the purple, red, and green boxes from A. Purple region shows a small droplet with a single bacterium present, which is highlighted by the cyan box. Red region shows a droplet in the upper right that is too thick to image through directly. Green region shows an ideal droplet with numerous bacteria present as indicated by white arrows.

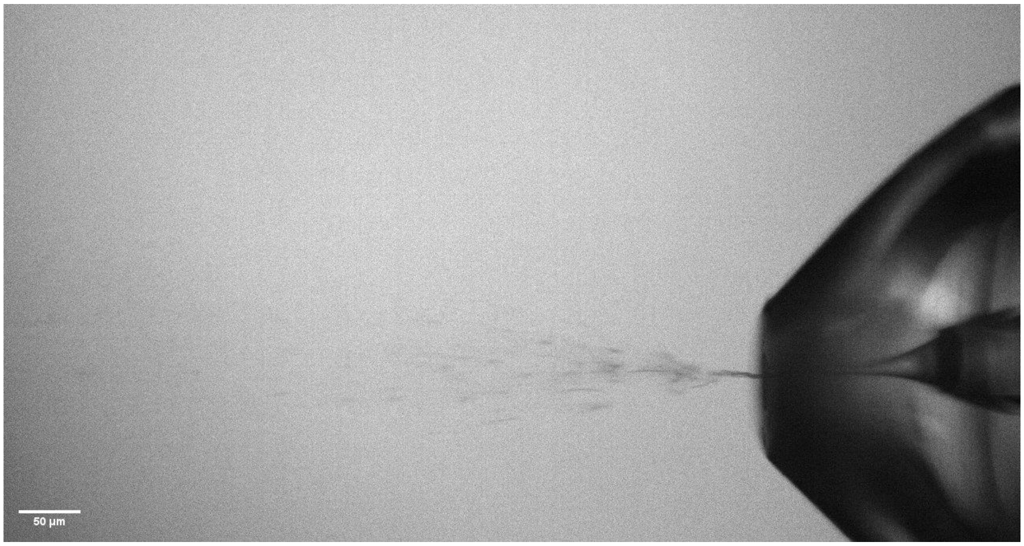

Figure S3: Imaging of the nozzle producing a spray with pulsed back light illumination. The total liquid flowrate is 55 µL/min and the nitrogen gas flowrate is 70 mg/min. A short jet rapidly breaks up into a heterogeneous and divergent spray.

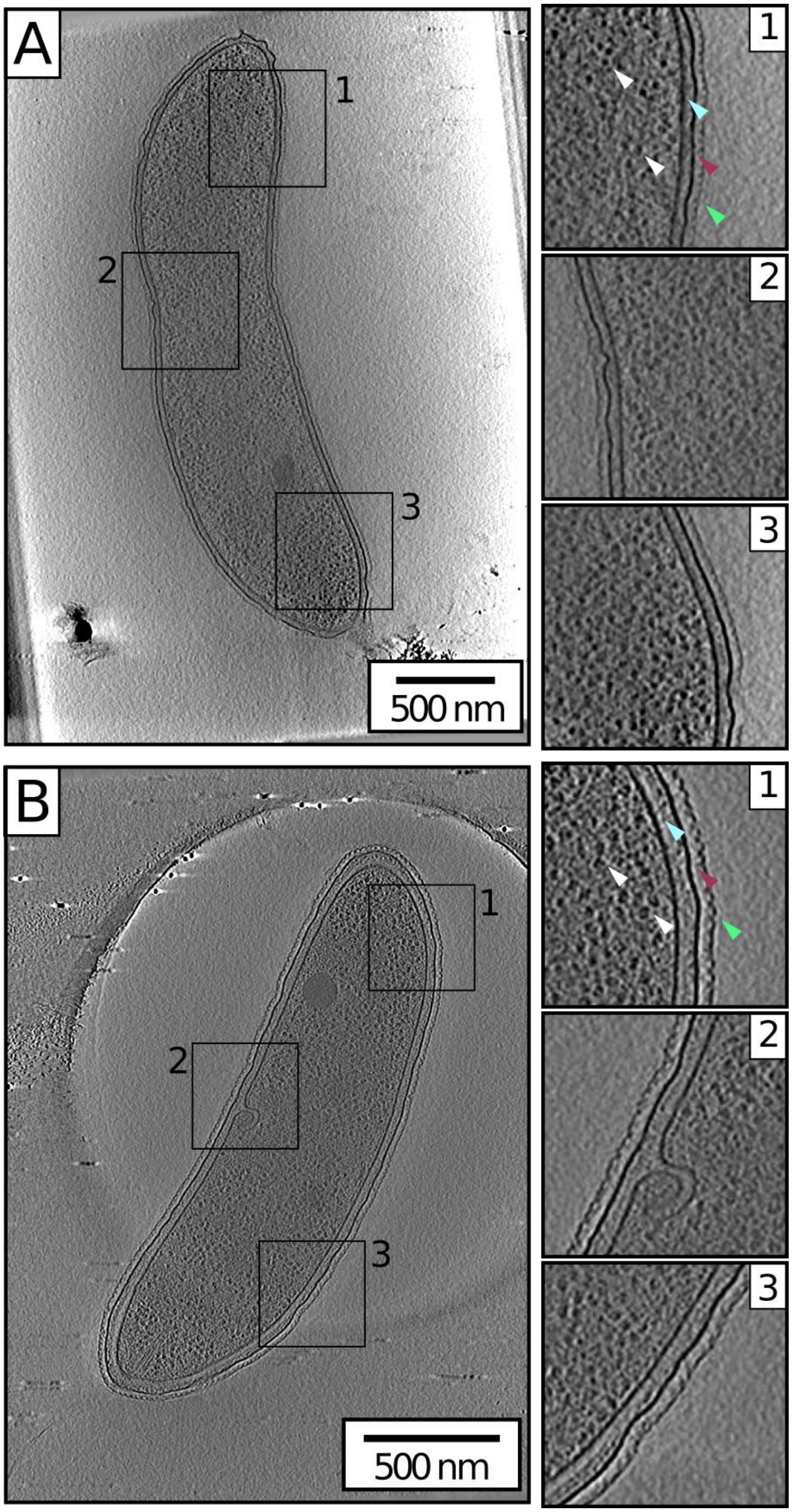

Figure S4: Representative tomography data from control cells mixed with growth media before spray deposition onto a grid (A) or prepared by traditional plunge freezing techniques (B). Each large image is a single slice from the tomographic reconstruction. Boxes highlight zoomed regions where the S-layer (green pointer), outer membrane (maroon pointer), inner membrane (cyan pointer), and ribosomes (white pointers) are clearly visible. Control cells from both conditions appear qualitatively similar.

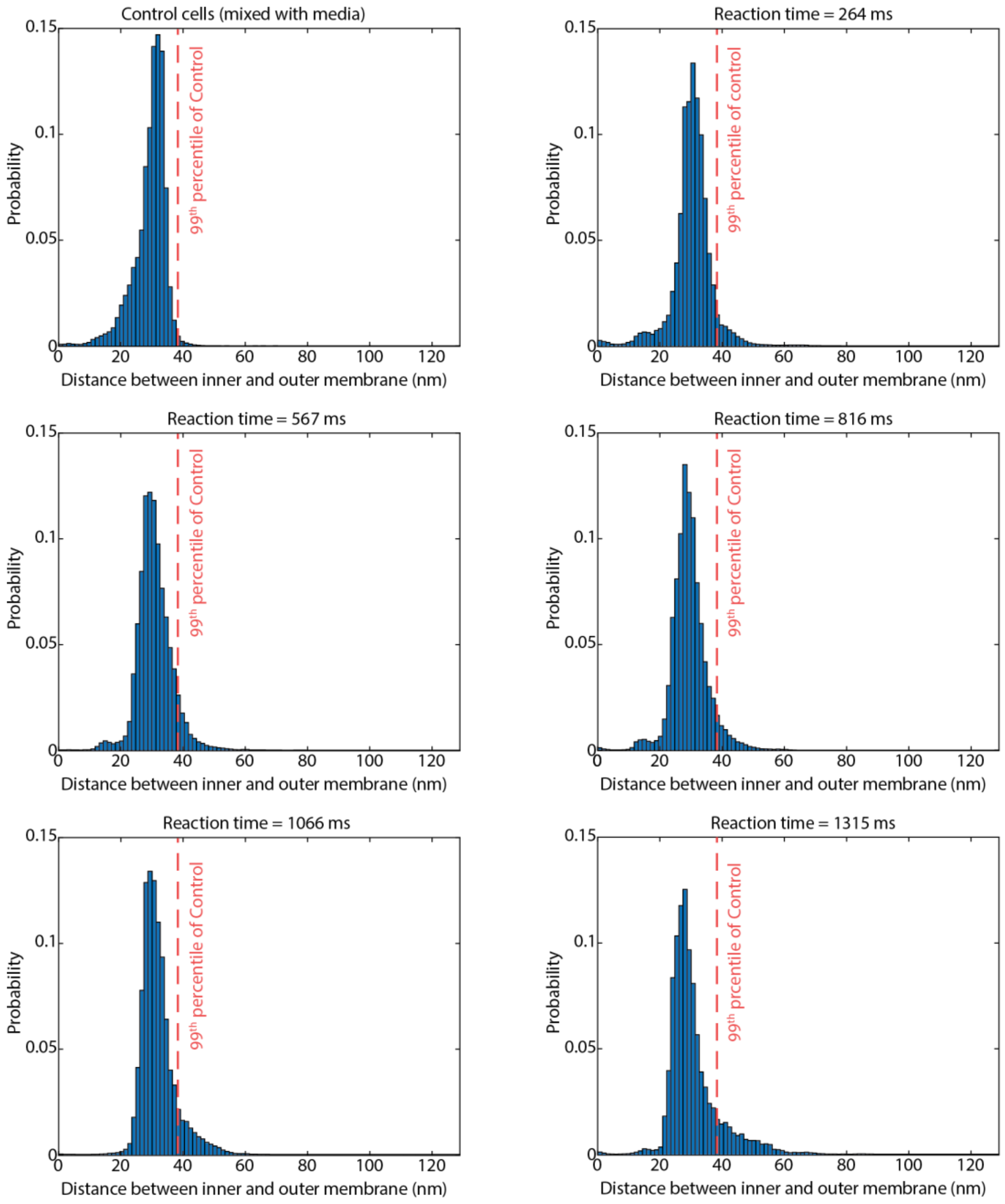

Figure S5: Complete histograms of the separations between inner and outer membrane for all the cells at the various reaction times observed. The cumulative probability greater than the dashed red line is shown in Figure 4C of the main text.

Mixer Geometry	Sample Flowrate (µL/min)	Sheath Flowrate (µL/min)	Mixer: Average Age at Complete Mixing ± Uncertainty (ms)	Mixer: Delay Time ± Uncertainty (ms)	Total Mixer: Timepoint ± Uncertainty (ms)	Time of Flight: Time (ms)	Plunge: Time ± Uncertainty (ms)	Freezing: Time (ms)	Final Timepoint ± Uncertainty (ms)
100 µm ID, 32 mm length	15.0	22.4	39 ±17	161 ± 11	200 ± 28	1	63±4	<1	264 ± 32
100 µm ID, 25 mm length; 320 µm ID, 17 mm length; 100 µm ID, 16 mm length	19.2	110.7	14 ± 6	486 ± 11	500 ± 17	4	63±4	<1	567 ± 21
100 µm ID, 25 mm length; 320 µm ID, 17 mm length; 100 µm ID, 16 mm length	17.8	85.1	17 ± 7	733 ± 22	750 ± 29	3	63±4	<1	816 ± 33
100 µm ID, 25 mm length; 320 µm ID, 17 mm length; 100 µm ID, 16 mm length	16.5	59.5	21 ± 9	979 ± 39	1000 ± 48	3	63±4	<1	1066 ± 52
100 µm ID, 25 mm length; 320 µm ID, 17 mm length; 100 µm ID, 16 mm length	15.1	34	30 ± 13	1220 ± 79	1250 ± 92	2	63±4	<1	1315 ± 96

Table S1: Mixer design and detailed contributions of the five distinct steps to the final time point probed. The "mixer: timepoint ± uncertainty" column is simply a sum of the "mixer: average age at complete mixing ± uncertainty" and "mixer: delay time ± uncertainty" columns. For full mixer definitions and calculations see Reference 1. For timepoints 567-1315 ms, a mixer with a three-part delay line was utilized. Specifically, it has an expansion region, with a larger inner diameter, to reduce the velocity of the sample to accrue a longer delay time without making the device overly long. The length and inner diameters of the three components of the delay region are specified in the table.

Discussion of timepoint uncertainty:

Multiple factors contribute to the reported reaction time of the frozen sample and its uncertainty. In order, the final reaction time is determined by five sequential processes: 1) the mixing, 2) delay times associated with the mixer, 3) the time of flight from the end of the nozzle to the grid, 4) the plunge time of the grid (with sample) into the ethane cup, and finally, 5) the freezing time. Each is briefly discussed below and values for each contribution for every timepoint are presented in Table S1.

1.) **Mixing time uncertainty:** Full details of the mixer timepoint calculation have been published previously[1]. In short, the time required for the stimulant, H_3O^+ ions in this case, to diffuse into the sample stream is calculated, and the system becomes 'fully mixed' when a certain threshold concentration is reached at the center of the sample stream. At this point an average age (and

standard deviation) of the full sample stream is determined (see column "mixer: average age at complete mixing ± uncertainty" in Table S1). This process typically occurs over only a short portion of the delay line, and can range from single to tens of milliseconds depending on the desired final timepoint. For timepoints 567-1315 ms, a mixer with a three-part delay region was used (as detailed in Table S1). For this design, the goal is to reach a fully mixed system quickly, before entering the delay region, which helps to keep the overall mixing uncertainty low.

2.) **Delay time during the flow in the capillary**: As the sample travels through the remaining portion of the mixer capillary, it continues to age. These distances are carefully considered to ensure that the proper time interval has passed, so that the desired time point is reached. The parabolic flow profile, or the spread of flow speed, is used to calculate the uncertainty for this delay time (see column "mixer: delay time ± uncertainty" in Table SI). In the case of the mixer with the expansion region (timepoints 567 – 1315 ms), the velocity and parabolic flow profile for each region of the mixer are calculated. The larger diameter of the expansion region leads to a wider spread in the flow speeds, creating a larger delay uncertainty, but this is an important tradeoff to reach longer timepoints while avoiding unpractically long delay lines. This does, however, dominate the uncertainty for these timepoints. Overall, the final timepoint at the exit of the mixer, as well as the overall uncertainty, is determined both by the mixing and delay times.

3.) **Time of flight**: The time of flight was approximated with jet speed calculations under jetting conditions, using the volumetric flow rate together with the (measured) jet diameter to compute the average flow speed. Then, the travel time for the 7 cm distance from nozzle tip to the grid was calculated. This estimate acts as an upper limit for the time of flight since the spray speed will be faster than the jetting speed because the flow rate of the accelerating gas is increased to create the spray. With jet speeds on the scale of 25 – 75 m/s, the longest time of flight is expected to be on the single millisecond scale (1 – 4 ms).

4.) **Plunging time and uncertainty:** Despite the mechanical motion being highly reproducible, there is an uncertainty in the plunging time of several ms due to the physical extent of the aerosol that is formed 7 cm away from the mixer. The aerosol produces a spread of droplets roughly 1 cm in diameter. The droplets deposited on the grid could come from the top or the bottom of this aerosol spread. If the aerosol is positioned at the very top of the accessible path of the grid (longest plunging time), the velocity of the plunger is ~1 m/s. This results in an uncertainty of 10 ms from top to bottom of the aerosol or ± 5 ms. If the aerosol is positioned at the very bottom of the accessible path of the grid (shortest plunging time), the velocity is ~1.6 m/s. This results in an uncertainty of ± 3 ms.

5.) **Freezing time and uncertainty:** The exact timescale of freezing is difficult to determine. Some of the most direct experimental evidence has involved the plunging of small thermocouples[2]. The observed cooling rates were ~6-7×10^4 K/s, suggesting cryogenic temperatures are reached within one millisecond. These cooling rates will depend on sample thickness and grid type but should be negligible for the time ranges investigated here.

	Control cells	t_{Rxn} = 264 ms	t_{Rxn} = 567 ms	t_{Rxn} = 816 ms	t_{Rxn} = 1066 ms	t_{Rxn} = 1315 ms	total
Tilt series	7	9	5	5	7	4	37
Tomograms used for analysis	5	8	5	5	6	4	33

Table S2: Statistics for the number of tilt series collected and the number of tomograms used for analysis. All data excluded from analysis was due to poor reconstruction quality.

References

1 Calvey, G. D., Katz, A. M. & Pollack, L. Microfluidic Mixing Injector Holder Enables Routine Structural Enzymology Measurements with Mix-and-Inject Serial Crystallography Using X-ray Free Electron Lasers. *Analytical Chemistry* **91**, 7139-7144, doi:10.1021/acs.analchem.9b00311 (2019).

2 Bikker, B. *Measurement of cooling rate during plunge freezing of sample preparation in cryo electron microscopy* Masters thesis, Delft University of Technology, (2019).
